# Defect-Induced Tunable Permittivity of Epsilon-Near-Zero in Indium Tin Oxide Thin Films

**DOI:** 10.3390/nano8110922

**Published:** 2018-11-07

**Authors:** Jiqing Lian, Dawei Zhang, Ruijin Hong, Peizhen Qiu, Taiguo Lv, Daohua Zhang

**Affiliations:** 1 Engineering Research Center of Optical Instrument and System, Ministry of Education and Shanghai Key Lab of Modern Optical System, University of Shanghai for Science and Technology, Shanghai 200093, China; LianJiqing1990@163.com (J.L.); rjhongcn@163.com (R.H.); qiupeizhen@126.com (P.Q.); lvtaiguo@lcu.edu.cn (T.L.); 2 School of Electrical and Electronic Engineering, Nanyang Technological University, Singapore 639798, Singapore; edhzhang@ntu.edu.sg

**Keywords:** ITO thin films, Epsilon-Near-Zero, defect, optical modulation

## Abstract

Defect-induced tunable permittivity of Epsilon-Near-Zero (ENZ) in indium tin oxide (ITO) thin films via annealing at different temperatures with mixed gases (98% Ar, 2% O_2_) was reported. Red-shift of λ_ENZ_ (Epsilon-Near-Zero wavelength) from 1422 nm to 1995 nm in wavelength was observed. The modulation of permittivity is dominated by the transformation of plasma oscillation frequency and carrier concentration depending on Drude model, which was produced by the formation of structural defects and the reduction of oxygen vacancy defects during annealing. The evolution of defects can be inferred by means of X-ray diffraction (XRD), atomic force microscopy (AFM), and Raman spectroscopy. The optical bandgaps (E_g_) were investigated to explain the existence of defect states. And the formation of structure defects and the electric field enhancement were further verified by finite-difference time domain (FDTD) simulation.

## 1. Introduction

Searching for better plasmonic materials was of great significance for the application of various ultrasensitive plasmonic devices [1,2,3,4]. However, most of the current plasmonic materials like noble metals exhibit limited tunability and feature high extinction losses in the range of visible and NIR spectra [5,6,7,8,9,10]. In recent years, transparent conductive oxides (TCOs) have been chosen as alternative plasmonic materials that include ITO, Al- or Ga-doped Zn oxides (AZO/GZO), and a mixed family of these oxides [11]. As a result of the absence of interband transitions, the imaginary part of dielectric constant of TCOs is smaller than that of noble metal [12]. Since TCOs can be heavily doped, their carrier concentration will be accordingly changed to tune the optical properties of them [13]. Furthermore, the dielectric constant of TCOs can be flexibly tuned and the Epsilon-Near-Zero (ENZ) behavior has been achieved in a wide range of spectrum [14,15,16]. According to the above, plasmonic TCOs (especially ITO) have been widely used in plasmonics, nonlinear optics, and metamaterials, such as perfect absorption [17,18,19], electro-optical modulation [20,21,22], switching [23,24,25], beam steering [26], nonlinear generation enhancement [14,15], and negative refraction [27,28,29].

A number of studies have mirrored that post-annealing treatment can tune the structural properties of ITO thin films which were deposited on different substrates [30,31,32], by controlling the gaseous atmospheres [33,34] or annealing temperature [35,36,37]. The electrical properties like free-carrier concentration have also been modulated combined with the change of the optical properties even the ENZ characteristics [38,39,40]. When it comes to the internal carrier transport of ITO films, many scattering mechanism were researched such as oxygen vacancy. In vacancy, doping of tin atom can cause ionized impurities, grain barriers, dislocation defect, and crystallographic defects [41,42,43,44]. What is more, other researchers adopted a post-annealing procedure to modify optical-electrical properties [45,46]. There are a few of reports concerning ENZ characteristics in NIR spectral ranges of ITO thin films which grow on K9 glass substrates by electron beam (EB) evaporation method.

In this work, a tunable permittivity of ENZ in indium tin oxide (ITO) thin films was realized via annealing. The surface morphology and crystallinity of samples were investigated at the beginning. Then, in order to characterize the dielectric and spectral properties of the films, we measured the permittivity and absorption spectra. To describe the mechanism of tunable permittivity, the Drude model was introduced. Meanwhile, the plasma frequency, carrier relaxation time, and optical bandgaps were theoretically and experimentally researched to explain the changes of optical properties [47,48,49]. Furthermore, the oxygen vacancy defect states and vibrational levels within ITO films were analyzed by Raman scattering spectra. At last, we verify the enhancement of absorption and the evolution of internal defects in the films by FDTD (finite-difference time domain) solutions.

## 2. Materials and Methods

Before deposition, the K9 glass substrates were ultrasonically cleaned in acetone, ethanol, and deionized water for 20 min, respectively, and then dried with a nitrogen flow. ITO thin films were grown by electron beam (EB) evaporation from ITO coating materials (90 wt.% In_2_O_3_ and 10 wt.% SnO_2_ target, 99.99% purity) with baking temperature set at 370 °C and a base pressure less than 5 × 10^−4^ Pa. The target thicknesses of 80 nm were monitored by a quartz crystal microbalance. All substrates were place on the fixture with the same radius away from the axis to ensure the uniformity of the film thickness.

Postdeposition annealing treatments had been carried out using a high temperature tubular furnace (SG-GL 1200K, Shanghai, China). All the ITO films annealing in mixed gases (98% Ar, 2% O_2_) were maintained for 60 min at three target temperatures of 150 °C, 300 °C, and 450 °C before a 5 °C per minute heating rate performed during the procedure under normal pressure. For comparison, an as-deposited film on bare substrate was grown in our work. As-deposited film and as-annealed films were indicated as S0, S1 (150 °C), S2 (300 °C), and S3 (450 °C) respectively.

The structural properties and the crystallinity of the films were measured by a Bruker AXS/D8 Advance X-ray diffraction (XRD) system (Bruker, Billerica, MA, USA) with Cu kα radiation (λ = 0.15408 nm). The surface morphology and roughness were characterized by AFM (atomic force microscopy) (XE-100, Park System, Suwon, Korea). The optical absorption was measured with an UV-Vis-NIR double beam spectrophotometer (Lambda 1050, Perkins Elmer, Waltham, MA, USA). The sheet resistances of films were measured by four-point probe resistivity measurement system. Hall measurements (HMS-3000, ECOPIA, Anyang, Korea) were conducted to determine the electron mobility, sheet resistance, resistivity, and free carrier concentration of the films. The dielectric constant characteristics were taken from spectroscopic ellipsometry (UVISEL-ER, HORIBA, Oberursel, Germany). The Raman spectrum was carried out by a confocal microprobe Raman system (inVia Raman Microscope, Renishaw, Pliezhausen, Germany) with a 633 nm operation wavelength. The analysis of the data used the finite-difference time domain (FDTD).

## 3. Results and Discussion

### 3.1. Microstructural Properties and Surface Morphology

**Figure 1:**
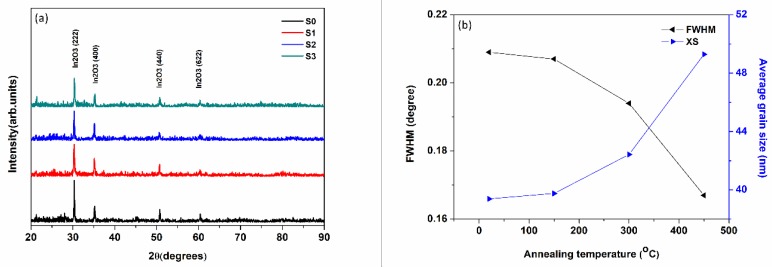
(**a**) The XRD intensities of as-deposited and as-annealed ITO films; (**b**) full-width at half-maximum (FWHM) and calculated average grain size of indium tin oxide (ITO) films as a function of annealing temperature in (222) crystallographic plane.

**Figure 2:**
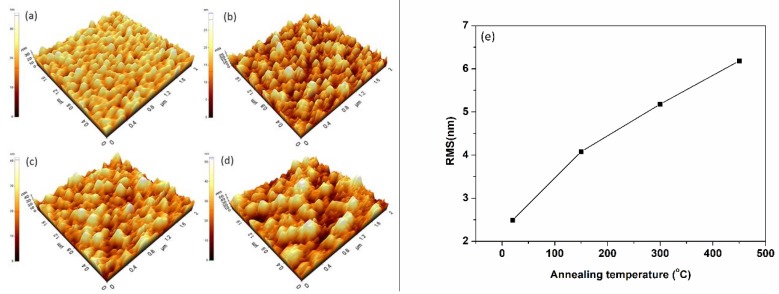
(**a**–**d**) AFM surface morphology patterns of ITO films before and after annealing. (**a**) for S0 (as deposited), (**b**) for S1, (**c**) for S2, and (**d**) for S3. (**e**) RMS values of ITO film annealing in different temperatures.

Figure 1a shows the XRD patterns which reveal the crystallinity of the samples. All the ITO films present the preferential orientation at approximately 30.58° (2θ) which corresponds to the (222) crystallographic plane of ITO according to the standard card of ITO (JCPDS: 06-0416). Other peaks appear at 2θ = 35.47° (400), 2θ = 51.04° (440), and 2θ = 60.68° (622). It is known that the growth orientation is biased towards the lowest surface energy. During the annealing procedure, the In charge is melted with the broken portion of the crystallographic texture. Parts of the atoms and so-called defects were evaporated and the others migrate through the substrate from the upper surface state to the lower due to the lowest principle of energy [50]. At the same time, all of these defects or atoms will annihilate and agglomerate within the internal structure resulting in recrystallization. This process constantly occurs in the structural transformation of ITO at the temperature range. However, the grain size is increased slightly during the annealing process. The recrystallization procedure can be deduced from both XRD (Figure 1b) and AFM results (Figure 2).

The average grain size is calculated by Scherrer formula, using FWHM (full-width at half-maximum) values of the XRD diffraction peaks as follows [51] D=0.9λ/(βcosθ), where D is the grain size, λ (=1.5406 Å) is the wavelength of X-ray radiation, θ the diffraction angle, and β is the FWHM which is obtained from MDI Jade analysis. The average grain sizes of S0, S1, S2, and S3 are 39.391, 39.764, 42.43, and 49.286 nm, respectively (Table 1). We can also see that the d-value (interplanar spacing) of the films exhibit a difference due to the change of residual stress. The diffraction peaks are 30.320°, 30.360°, 30.341°, and 30.417° (2θ), respectively. The FWHM values of S0, S1, S2, and S3 are 0.209°, 0.207°, 0.194°, and 0.167°, respectively. Figure 1b points out that the average grain size and the FWHM are related to the annealing temperature in (222) crystallographic plane. With the temperature increasing, the grain size is increased slightly. The FWHM of the as-annealed samples was narrow than that of the as-deposited sample. The results consist of the broken of the crystallographic texture and the agglomeration of the grain (as shown in Figure 2a–d).

Figure 2 reveals the AFM images and the root-mean-square surface roughness (RMS) of samples as a function of annealing temperatures with the scanning area of 2 × 2 μm. Obviously, the surface morphology of as-deposited film is uniform and well-distributed so that the value of RMS is relatively small. When annealing at 150 °C, the film turns into valley-like and crisscross along with a large grain size consisted with Figure 1. When the temperature increases to 300 °C or 450 °C, the surface transforms from smooth to ups and downs. Owing to the local thermal effect and the increased heat transferring from furnace to the films, the interior recombination of intrinsic atoms and defects occurs leading to grain growth. This is also consistent with the XRD results which show a better crystalline of the samples after annealing. What is more, the measured RMS values consist with the surface patterns in Figure 2. All these changes were attributed to the increased grain size and induced defects [35].

### 3.2. Optical Constants

#### 3.2.1. Optical Permittivity

**Figure 3:**
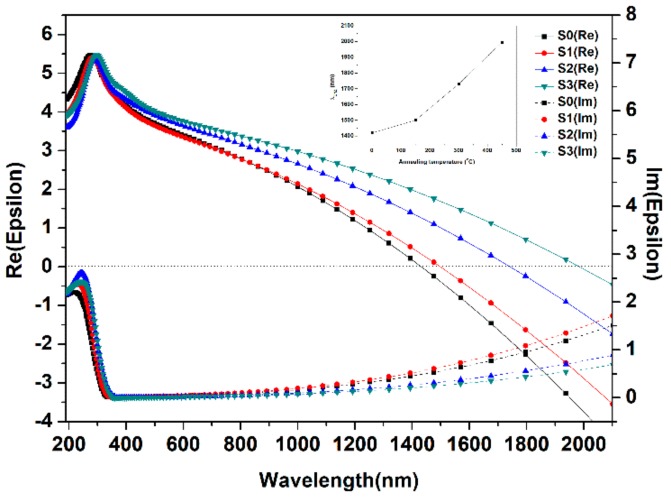
Real (solid line) and imaginary (dash line) part of permittivity of ITO films before and after annealing. The inset shows λ_ENZ_ as a function of annealing temperature.

The optical permittivity ε(ω) of ITO films was investigated by spectroscopic ellipsometry method. Figure 3 expresses the measured permittivity which illustrates the influence of the annealing procedure on the real (ε) and imaginary (ε) parts of ε(ω). From Figure 3 and the inset figure, ITO films show an ENZ condition which is denoted by λ_ENZ_ [52,53]. The λ_ENZ_ of ITO films are 1422 nm (S0), 1504 nm (S1), 1730 nm (S2), and 1995 nm (S3), respectively. Accordingly, when the ENZ wavelength (λ_ENZ_) shifts to red and the imaginary permittivity reduces to less than 1 with increasing temperature. Furthermore, the real permittivity is nearly zero while the imaginary part is smaller than 1 which guarantees the internal fields enhancement that supports the ENZ condition in ITO films.

The Drude–Sommerfeld model is adequately introduced to describe the mechanism of the optical constants of ITO film [13,49]:

(1) $$\varepsilon_{\mathrm{ITO}}\left(\omega \right)=\varepsilon_{1}\left(\omega \right)+{i\varepsilon }_{2}\left(\omega \right)=\varepsilon_{\infty }-\frac{\omega_{p}{}^{2}}{\omega^{2}+i\Gamma \omega }\;$$

Here, ε_∞_ is the high frequency limit of ε_ITO_, ω_p_ is the plasma frequency, and Γ expresses the charge carrier collision rate, which leads to the optical losses ε_2_ inside the film. The plasma frequency can be described as

(2) $$\omega_{p}{}^{2}=\frac{{\mathrm{ne}}^{2}}{\varepsilon_{0}m^{*}}\;$$

where n is the carrier concentration, ε_0_ is the permittivity of free space, e is electron charge, and m* is the effective mass of electron, respectively. Generally, for the sake of negative permittivity, a large free carrier concentration with the order of 10^20^ to 10^22^ cm^−3^ is needed. The intrinsic doping is so large in ITO film that brings about the ENZ characteristic in the NIR region. Therefore, ITO films exhibit metal behavior in the NIR range [10,13]. We can learn from Equations (1) and (2) that the plasma frequency, charge carrier collision rate, and carrier concentration may alter the optical permittivity of the films. It will be discussed in the following sections.

#### 3.2.2. Optical Bandgap and Optical Absorption

**Figure 4:**
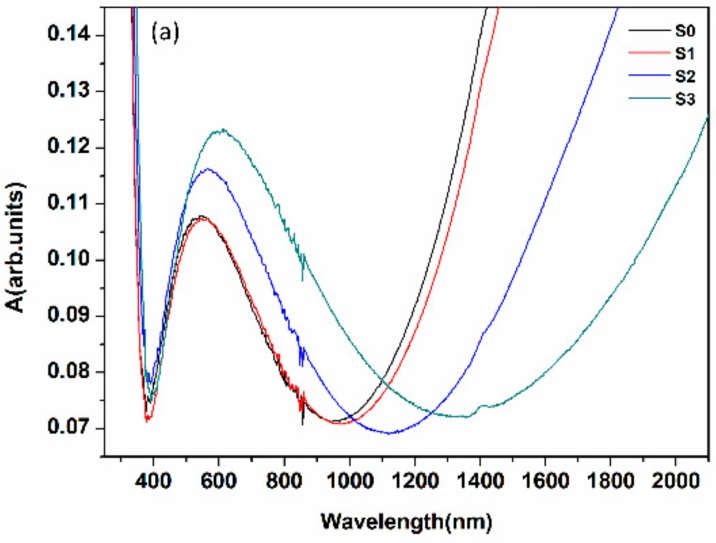

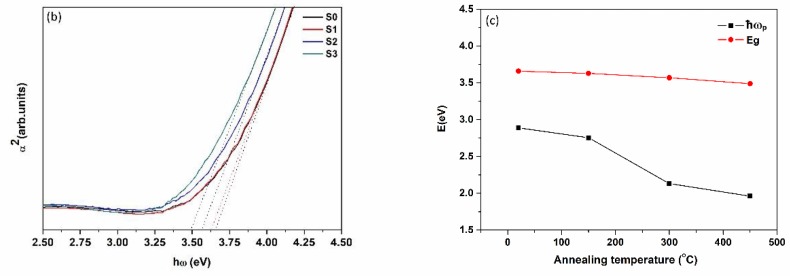
(**a**) The optical absorption of ITO as a function of wavelength; (**b**) α^2^ versus hω curves for the optical band gap determination in the samples; and (**c**) the calculated ħω_p_ and extrapolated E_g_ as a function of annealing temperature (ω_p_ was the calculated plasma frequency from Figure 5).

Here, the optical absorption was measured first to check the effects of structure defects. Meanwhile, the optical bandgap shift was experimentally calculated using Tauc’s method to learn the relationship between the oxygen vacancies and the bandgap properties of ITO films [47,48]. As shown in Figure 4a, the absorption in visible enhances and redshifts but dropped off in NIR. It can be interpreted as the light trapping effect promoted by the rough structure defects [54,55]. Figure 4b shows that the bandgap narrows after annealing. It can be inferred that the defect energy levels generated by structure and oxygen vacancy defects act as the intermediate level. When the photon energy is applied to the films, electron transitions were taken place from valence band to defect energy level and then to the conduction band [42,56,57]. Consequently, lower energy is needed during the transition progress consistent with the red shift of absorption and the narrowing of bandgap.

Theoretically, it is clear that

(3) $$A=1-\mathrm{lgT},\;\;\;T=\left(1-R\right)e^{-\alpha d}\;$$

where A is optical absorbance, α is absorption coefficient, T is optical transmittance, R is the total reflectance coefficient, and d represents the film thickness. The optical bandgap E_g_ for a direct-gap semiconductor can be described by a function of frequency ω:

(4) $$\;\alpha ћ\omega ={\left(ћ\omega -E_{g}\right)}^{1/2}\;$$

According to Burstein–Moss shift effect, the bandgap E_g_ has a connection with carrier concentration n, which is described as [58]

(5) $$E_{g}=E_{g0}+\frac{{ћ}^{2}}{2m^{*}}{\left(3\pi^{2}n\right)}^{2/3}\;$$

Here, E_g0_ is the intrinsic bandgap, n is the carrier concentration, and m* indicates the effective mass of electron (here m* of ITO is an approximately constant: 0.38m_0_ [59,60,61]).

Based on the formula above, while the annealing temperature rises, the bandgap E_g_ decreases due to the diminution of carrier concentration n. From Figure 4a it can be see that the optical absorption shows a red shift from 546 nm to 610 nm owing to the electron transition from the valence band to the defect energy levels, or from the defect to the conduction band [62,63,64]. Moreover, Figure 4c suggests the calculated ħω_p_ and extrapolated E_g_ as a function of annealing temperature. With the increase of temperature, the values are decreasing, but the difference between them is more obvious. We calculate the absorption coefficient by Formula (4), catching an upward tendency which is consistent with Figure 4a. Finally, all of the variation is owing to the internal carrier fundamentally changes which root in the recombination of internal structure.

### 3.3. Electrical Properties

To explore the electrical mechanism, the bulk carrier concentration n, sheet carrier concentration n’, the electron mobility μ, and resistivity ρ were measured by Hall Effect measurement. Meanwhile, we calculated the plasma frequency ω_p_, carrier relaxation time τ and sheet resistance Rs’ by Equation (2) combined with the following formula.

(6) $$\mu =\left(\tau e\right)/m^{*},\;\;\;n=m^{*}/\left({\rho \tau e}^{2}\right)\;$$

(7) $$\;\rho =R_{s}^{\prime }d\;,\;\;\;n=\;n\prime /d\;$$

**Figure 5:**
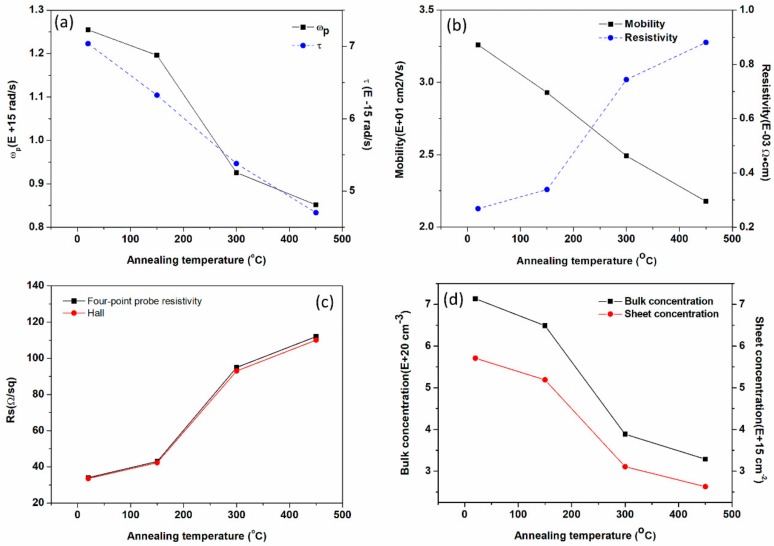
(**a**) The calculated plasma frequency ω_p_ and carrier relaxation time τ as a function of annealing temperature. (**b**) The resistivity ρ and electron mobility μ obtained from Hall Effect measurement. (**c**) The compared values of sheet resistance Rs measured by Hall Effect measurements and four-point probe resistivity measurement system. (**d**) The measured bulk carrier concentration n and sheet carrier concentration n’.

All of the electrical relevant data mentioned above are shown in Figure 5a–d. 

Figure 5 shows the reduction of the plasma frequency and carrier relaxation time (Figure 5a) due to the decrease of carrier concentration and mobility (Figure 5b) as the annealing temperature rise. During the annealing procedure, the structure defects in ITO films scatter the free carrier resulting in the reduction of mobility. The decrease of sheet/bulk carrier concentration (Figure 5d) is attributed to the oxygen vacancy reduction and the carrier trapping effect in the rough surface structure [41]. The resistivity (Figure 5c) of ITO films tested by the two methods fits well and fits with the evolution of carrier concentration and mobility. What is more, the downtrend of calculated ω_p_ and τ can also be certified by the red-shift of absorption spectra reckoned from Figure 5a–c. Equations (1)–(7) connect the dielectric properties and electrical performance of ITO in this work. These equations imply that $\varepsilon_{2}\propto \omega_{p}{}^{2}\propto n,\;\mu \propto \tau \propto n,{\;E}_{g}\propto n,\;A\propto (1/n),\;\rho \propto R_{s}\propto (1/n)\;i.e.$ It is easy to explain the tunability of the permittivity by the free carrier mechanism that has a key role in determining the plasma frequency ω_p_. This carrier mechanism is due to the defect modulation discussed earlier; it also infers that $\lambda_{\mathrm{ENZ}}\propto (1/n),{\;X}_{s}\propto \mathrm{RMS}\propto (1/n)\;i.e$.

### 3.4. Raman Spectra and FDTD Simulation

**Figure 6:**
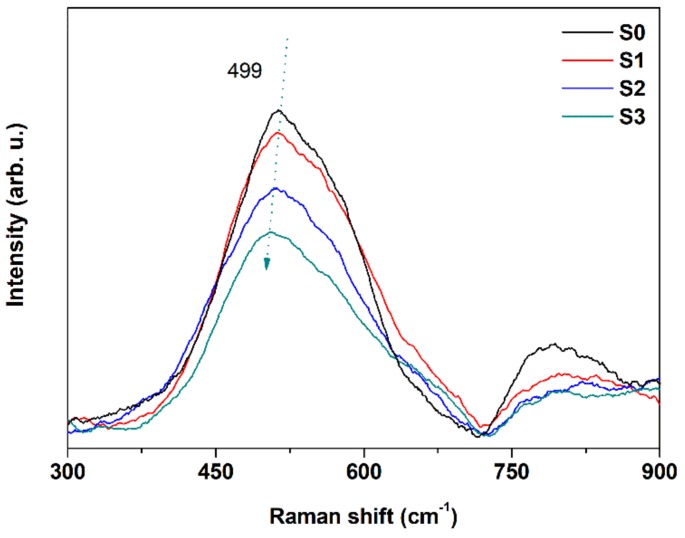
Raman spectra of the as-deposited and annealed ITO films.

The oxygen vacancy defect states and stretching vibrational levels within ITO films were researched by Raman scattering. ITO has been proven to have 22 Raman responsive and 16 infrared responsive modes. The 499 cm^−1^ mode is rooted in the bending and stretching vibrations of InO_6_ octahedra. The oxygen vacancy defect has a great influence on the vibration of In–O–In bond, and it also causes the change of Raman scattering intensity and the shift of Raman peak position since the scattering intensity is proportional to the square of the derivative of the polarizability [56]. Here, we attribute the change of Raman spectra intensity to the reduction of oxygen vacancies and the rough surface. According to Figure 6, high frequency sets at 499 cm^−1^ are typically assigned to bcc (body-centered cubic)-In_2_O_3_ [65,66,67,68]. The blue-shift and the decreased intensity of 499 cm^−1^ peak demonstrate that there are fewer oxygen vacancies in ITO films after annealing according [65]. It indicates that oxygen vacancies are partially filled during the annealing procedure [69]. The broken of In–O–In bond also can be confirmed by the dissociated and agglomerated rough structure.

**Figure 7:**
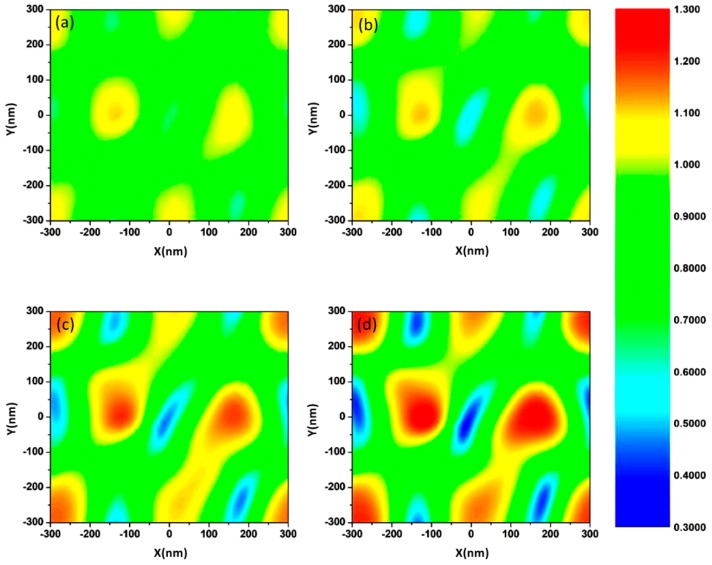
FDTD simulation patterns of electric field amplitude for ITO films: (**a**) S0, (**b**) S1, (**c**) S3, and (**d**) S4.

To further verify the absorption changes and the optoelectronic properties of the films, the electric field distribution on the rough surface was simulated by FDTD solutions. During the calculation, a 600-nm (consistent with the optical absorption peak wavelength) laser was irradiated perpendicular to the x-y plane of the ITO film and polarized along the y-axis. The values of different parameters including optical refractive index and RMS applied in the model are consistent with the experiment data. According to Figure 7a, the electric field is well-distributed and the enhancement effect is not significant due to the smooth surface. With the temperature increasing, the electric field intensity enhances and the density of free electrons begins to appear maldistributed. This result is consistent with the reduction of free carrier concentration owing to the increase of internal structure defects and the decrease of oxygen vacancies. The surface “hot spots” (as shown in Figure 7b–d) verify the enhancement of absorption. The defects in the ITO thin film enhanced the localized surface electric field and visible absorption intensity. The simulation results fit well with the experimental evidence.

## 4. Conclusions

In conclusion, the photoelectric properties of single-layer ITO thin films were modulated by a simple post-annealing method. Red-shift of λ_ENZ_ (Epsilon-Near-Zero wavelength) from 1422 nm to 1995 nm in wavelength was achieved. XRD and AFM results indicated that structure defects were induced and oxygen vacancies were filled during the annealing procedure that led to the degradation of free carrier concentration and mobility. The reduction of plasma frequency prompted the red shift of absorption with the decreased optical losses. The bandgaps were narrowed with the generation of defect levels. Raman scattering demonstrated the reduction of oxygen vacancies. FDTD solutions further verified the enhanced electric field distribution and optical absorption. It is possible to tailor the characteristics of ITOs through appropriate postdeposition treatment; making widely tunable ENZ ITO films will open up new opportunities in optoelectronic applications.

## Figures and Tables

**Table 1:** XRD results of θ–2θ scans for the films before and after annealing at different temperatures.

Sample	Annealing Temperature (°C)	2θ (°)	FWHM (°)	Interplanar Spacing d (nm)	Average Grain Size (nm)
S0	0 (As-deposited)	30.417	0.209	2.9363	39.391
S1	150	30.341	0.207	2.9435	39.764
S2	300	30.360	0.194	2.9417	42.430
S3	450	30.320	0.167	2.9454	49.286
